# Synthesis and bioactivities of 1-(4-hydroxyphenyl)-2-((heteroaryl)thio)ethanones as carbonic anhydrase I, II and acetylcholinesterase inhibitors

**DOI:** 10.3906/kim-2004-36

**Published:** 2020-08-18

**Authors:** Cem YAMALI, Halise İnci GÜL, Yeliz DEMİR, Cavit KAZAZ, İlhami GÜLÇİN

**Affiliations:** 1 Department of Pharmaceutical Chemistry, Faculty of Pharmacy, Atatürk University, Erzurum Turkey; 2 Department of Pharmacy Services, Nihat Delibalta Göle Vocational High School, Ardahan University, Ardahan Turkey; 3 Department of Chemistry, Faculty of Science, Atatürk University, Erzurum Turkey

**Keywords:** Carbonic anhydrases, acetylcholinesterase, heterocyclic, phenol

## Abstract

The discovery of enzyme targeting inhibitors is a popular area of drug research. Biological activities of the compounds bearing phenol and heteroaryl groups make them popular groups in drug design targeting important enzymes such as acetylcholinesterase (AChE, E.C.3.1.1.7) and carbonic anhydrases (CAs, EC 4.2.1.1). 1-(4-hydroxyphenyl)- 2-((aryl)thio)ethanones as possible AChE and CAs inhibitors were synthesized, and their chemical structures were confirmed by IR, ^1^H NMR, ^13^C NMR, and HRMS. The compounds 2 and 4 were found potent AChE inhibitors with the Ki values of 22.13 ±1.96 nM and 23.71 ±2.95 nM, respectively, while the compounds 2 (Ki = 8.61 ±0.90 nM, on hCA I) and 1 (Ki = 8.76 ±0.84 nM, on hCA II) had considerable CAs inhibitory potency. The lead compounds may help the scientists for the rational designing of an innovative class of drug candidates targeting enzyme-based diseases.

## 1. Introduction

Acetylcholinesterase (AChE, E.C.3.1.1.7) enzyme plays a vital role in the treatment of Alzheimer’s disease (AD). AChE is also one of the targets for several cholinergic toxicants, plant glycoalkaloids, and drug candidates [1–3]. Acetylcholine (ACh) is converted to choline and acetic acid molecules in synapses via the AChE enzyme. Modern AD therapy targets enhance cholinergic neurotransmission by way of AChE inhibitors (AChEIs). Widely used AChEIs are galantamine, donepezil, and rivastigmine [4,5]. However, these drugs are unfortunately less effective to block AD progression, and they also have side effects [6]. Also, the discovery of novel, effective, and therapeutic drug candidates targeting AChE is one of the widespread issues in medicinal chemistry.

Many heteroaromatic compounds such as benzoxazole [1], triazole [7], tetrazole [8], oxadiazole [6], and benzimidazol [9] which have the potency to make molecular interactions within the ChE enzyme were reported with their ChE inhibitory effects. In addition to these structures, the problematic process of AD directs scientists to search for new therapeutic multifunctional compounds [10,11]. Therefore, phenolic compounds might be a promising structure in the designing of novel AChEIs since the phenolic compounds exhibit cholinesterase inhibitory potency and antioxidant effects [12]. Recently, Sang et al. reported phenolic compounds as AChE inhibitors. In series, the compound, namely TM-3, showed a favorable AChE inhibitory effect with IC_50_ of 0.69 μM. Molecular docking studies also showed that a 2,4-dihydroxyacetophenone nucleus interacts with the enzyme via intermolecular hydrogen bonds [13].

**Figure 1:**
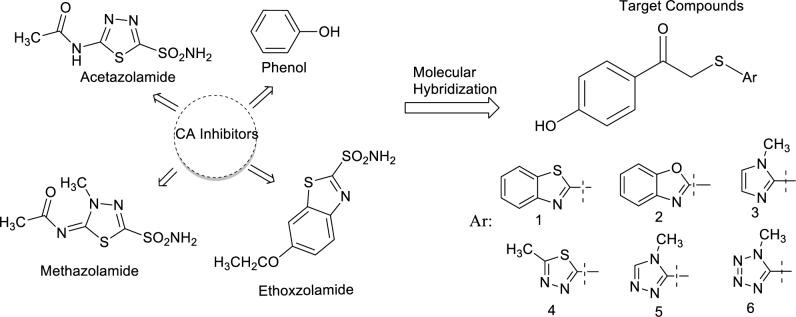
The design strategy of the target compounds.

Carbonic anhydrases (CAs, EC 4.2.1.1) are zinc bearing metalloenzymes which contribute to the controlling of pH balance by the acceleration of the hydration reaction of carbon dioxide in the living cells [14–16]. Regulation of CAs activity by using inhibitors is used in the clinic as antiglaucoma drugs and diuretics [17,18]. Besides, CAIs might have potential as anticancer, antiobesity, and antiinfective agents [19, 20]. To date, 15 α - CA isoforms in mammals have been reported. Among them, the cytosolic CA I and CA II isoforms are well-established targets for several diseases. While CA I is responsible for cerebral edema, CA II inhibition has a role for glaucoma, edema, altitude sickness, and epilepsy [21–23]. The sulfonamides and their bioisosteres (sulfamates, sulfamides, etc.) are the main pharmacophore groups which interact with the active site of CA isoenzymes [24]. For instance, sulfonamide having acetazolamide, methazolamide, ethoxzolamide (Figure 1) are widely used systemic antiglaucoma drugs in the clinic and these drugs have heterocyclic structures in their chemical skeleton. After that, the investigation of CA inhibition capacity and the mechanism of action of the phenol (Figure 1) led to design novel phenolic compounds targeting CA isoenzymes [25–27]. Hence, the substitution of phenol pharmacophore can be considered in the design strategy to have potent compounds with enhanced CA activity.

Based on the reports, different heterocyclic rings were combined with the phenol functional group since the phenol is one of the popular CAIs group and promising potential pharmacophore for new AChEIs. This study aimed to report CAs and AChE enzyme inhibitory potencies of the 1-(4-hydroxyphenyl)-2-((aryl)thio)ethanones 1–6 to find out CAs and AChE enzyme inhibitors for further studies.

## 2. Materials and methods

### 2.1. Chemistry

NMR spectra were recorded by Bruker AVANCE III 400 MHz (Bruker, Karlsruhe, Germany) and Varian Mercury Plus Spectrometer 400 MHz (Varianinc., Palo Alto, California, U.S.) in DMSO - *d6* (Merck KGaA, Darmstadt, Germany). LCMS - IT - TOF system (Shimadzu, Tokyo, Japan) was used for HRMS spectra. Electrothermal 9100 (IA9100, Bibby Scientific Limited, Staffordshire, UK) device was used to measure melting points (Mp). TLC - Silicagel HF254 (Merck Art 5715) plate was used to check the reaction process under UV lamb (Spectroline, ENF - 240C/ FE, New York, U.S.A). IR spectra of the compounds were recorded as potassium bromide pellets on a Perkin Elmer 100 Fourier transform (FT)-IR spectrophotometer (PerkinElmer, Inc., Waltham MA, USA)

### 2.2. A general synthesis method of the compounds 1–6 [28] (Figure 2)

Mercapto-based compounds (9.3 mmol) [Benzo[ *d* ]thiazole-2-thiol for 1 (1.6 g), 1 *H* - benzo[ *d* ]oxazole-2-thiol for 2 (1.43 g), 1-methyl-1 *H* -imidazole-2-thiol for 3 (1.1 g), 5-methyl-1,3,4-thiadiazole-2-thiol for 4 (1.23 g), 4-methyl-4 *H* -1,2,4-triazole-3-thiol for 5 (1.1 g), 1-methyl-1 *H* -tetrazole-5-thiol for 6 (1.1 g)] was dissolved at room temperature in fresh methanolic NaOH solution (0.4 g, 25 mL methanol) and stirred for 10 min. Then, 2-bromo-4’-hydroxyacetophenone (9.3 mmol, 2 g) was put into the reaction flask. After the final mixture was stirred for several hours at room temperature, it was taken into the water (50 mL). The white solid obtained was filtered, washed with water three times, and then dried. The compounds were purified by crystallization using suitable solvents such as ethanol (1–4, 6 ) and ethanol: DMF (5).

**Figure 2:**
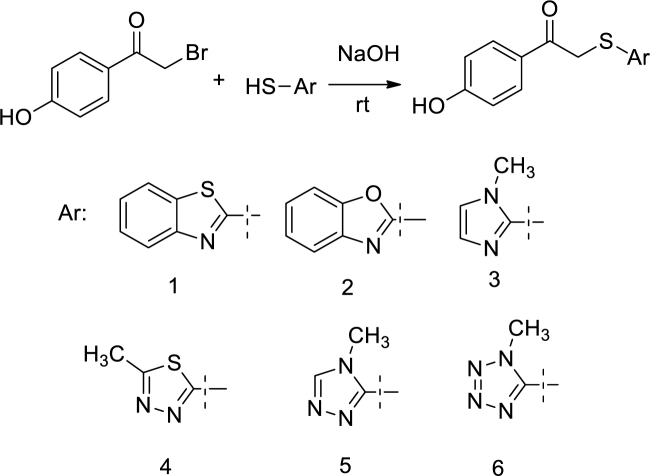
Synthesis of the compounds 1–6.

#### 2-(Benzo[ *d* ]thiazol-2-ylthio)-1-(4-hydroxyphenyl)ethanone, 1

Mp = 188–189 °C. Yield 72%. IR (KBr) cm^-1^: 3209, 3061, 3019, 2959, 2914, 1660, 1575, 1414, 1202, 1169, 993, 829, 753, 725. ^1^H NMR (400 MHz, DMSO - *d6* , δ , ppm) 7.99 - 7.93 (m, 3H, ArH), 7.76 (d, J = 7.7 Hz, 1H, ArH), 7.44 - 7.39 (m, 1H, ArH), 7.35 - 7.31 (m, 1H, ArH), 6.86 (d, J = 8.2 Hz, 2H, ArH), 5.06 (s, 2H, -CH2 -). ^13^C NMR (100 MHz, DMSO - *d6* , δ , ppm) 191.5 (C=O), 166.8, 163.4, 153.2, 135.4, 131.9, 127.5, 127.0, 125.1, 122.5, 121.7, 116.1, 41.4 (-CH_2-_). Predicted [M + H]^+^ 302.0304; measured [M + H]^+^ 302.0311.

#### 2-(Benzo[ *d* ]oxazol-2-ylthio)-1-(4-hydroxyphenyl)ethanone, 2

Mp = 210–212 °C. Yield 78%. IR (KBr) cm^-1^: 3051, 2982, 2937, 1668, 1579, 1474, 1451, 1201, 1172, 1146, 1000, 817, 750, 734. ^1^H NMR (400 MHz, DMSO - *d6* , δ , ppm) 10.5 (s, 1H, -OH), 7.94 (d, J = 8.6 Hz, 2H, ArH), 7.62 - 7.56 (m, 2H, ArH), 7.30 - 7.27 (m, 2H, ArH), 6.88 (d, J = 8.6 Hz, 2H, ArH), 5.07 (s, 2H, -CH_2-_). ^13^C NMR (100 MHz, DMSO - *d6* , δ , ppm) 191.1 (C=O), 164.7, 163.4, 151.9, 141.9, 131.8, 127.3, 125.3, 124.9, 118.9, 116.1, 110.8, 40.9 (-CH_2-_). Predicted [M + H]^+^ 286.0532; measured [M + H]^+^ 286.0537.

#### 2.2.3. 1-(4-Hydroxyphenyl)-2-((1-methyl-1H-imidazol-2-yl)thio)ethanone, 3

Mp = 214–215 °C. Yield 91%. IR (KBr) cm^-1^: 3125, 3101, 2947, 2991, 2806, 2680, 2610, 2499, 1655, 1585, 1460, 1277, 1263, 1253, 1130, 846, 772. ^1^H NMR (400 MHz, DMSO - ^d6^, δ , ppm) 10.5 (s, 1H, -OH), 7.80 (d, J = 8.8 Hz, 2H, ArH), 7.18 (d, J = 1.1 Hz, 1H, ArH), 6.88 (d, J = 1.1 Hz, 1H, ArH), 6.82 (d, J = 8.8 Hz, 2H, ArH), 4.49 (s, 2H, -CH_2-_), 3.53 (s, 3H, -CH_3_) . ^13^C NMR (100 MHz, DMSO - *d6* , δ , ppm) 192.6 (C=O), 162.9, 139.8, 131.5, 128.9, 127.2, 123.9, 115.8, 49.1 (-CH_2-_), 33.4 (-CH_3_). Predicted [M + H]^+^ 249.0692; measured [M + H]^+^ 249.0682.

#### 2.2.4. 1-(4-Hydroxyphenyl)-2-((5-methyl-1,3,4-thiadiazol-2-yl)thio)ethanone, 4

Mp = 200–202 °C. Yield 70%. IR (KBr) cm^-1^: 3036, 2948, 2912, 2804, 2688, 2608, 2500, 1655, 1573, 1499, 1389, 1296, 1198, 1173, 988, 829, 700. ^1^H NMR (400 MHz, DMSO - *d6* , δ , ppm) 10.5 (bs, 1H, -OH), 7.90 (d, *J* = 8.4 Hz, 2H, ArH), 6.86 (d, *J* = 8.4 Hz, 2H, ArH), 4.95 (s, 2H, -CH_2-_), 2.63 (s, 3H, -CH_3_). 13 C NMR (100 MHz, DMSO - *d6* , δ , ppm) 191.1 (C=O), 165.9, 164.9, 163.2, 131.6, 127.2, 115.9, 41.8 (-CH_2_2), 15.6 (-CH_3_). Predicted [M + H]^+^ 267.0256; measured [M + H]^+^ 267.0247.

#### 2.2.5. 1-(4-Hydroxyphenyl)-2-((4-methyl-4 *H* -1,2,4-triazol-3-yl)thio)ethanone, 5

Mp = 270–271 °C. Yield 67%. IR (KBr) cm^-1^: 3125, 3001, 2960, 2925, 2811, 2694, 2612, 2524, 1670, 1600, 1523, 1460, 1289, 1204, 1174, 826, 695. ^1^H NMR (400 MHz, DMSO - *d6* , δ , ppm) 8.52 (s, 1H, ArH), 7.85 (d, *J* = 8.8 Hz, 2H, ArH), 6.84 (d, *J* = 8.8 Hz, 2H, ArH), 4.73 (s, 2H, -CH_2-_), 3.57 (s, 3H, -CH_3_). ^13^C NMR (100 MHz, DMSO - *d6* , δ , ppm) 191.9 (C=O), 163.1, 149.2, 146.6, 131.6, 127.1, 115.8, 41.1 (-CH _2-_ ), 31.25 (-CH_3_). Predicted [M + H] ^+^ 250.0645; measured [M + H]^+^ 250.0634.

#### 2.2.6. 1-(4-Hydroxyphenyl)-2-((1-methyl-1 *H* -tetrazol-5-yl)thio)ethanone, 6

Mp = 199–201 °C. Yield 74%. IR (KBr) cm^-1^: 3108, 3031, 2981, 2934, 2818, 2695, 2614, 2542, 1672, 1586, 1383, 1293, 1209, 1185, 1014, 825, 700. ^1^H NMR (400 MHz, DMSO - *d6* , δ , ppm) 10.5 (bs, 1H, -OH), 7.89 (d, *J* = 8.8 Hz, 2H, ArH), 6.86 (d, *J* = 8.8 Hz, 2H, ArH), 4.98 (s, 2H, -CH_2-_), 3.97 (s, 3H, -CH_3_). ^13^C NMR (100 MHz, DMSO - *d6* , δ , ppm) 191.0 (C=O), 163.3, 153.9, 131.6, 126.9, 115.9, 41.7 (-CH_2-_), 34.1 (-CH_3_). Predicted [M + H]^+^ 251.0597; measured [M + H]^+^ 251.0588.

### 2.3. AChE inhibition assay

AChE inhibitory effects of the newly synthesized compounds were utilized by Ellman’s test [29] as previous studies with minor modifications [30–32]. Briefly, sample solution (750 μL) dissolved in deionized water with Tris/HCl buffer (100 μL, 1 M, pH = 8) at different concentrations, and then AChE solution (50 μL) was added. The final mix was incubated at 21 °C for 64 min. The reaction was initiated by acetylthiocholine iodide (50 μL). Then, 5,5′ -dithio-bis(2-nitro-benzoic)acid (50 μL, 0.5 mM) was added. The hydrolysis process was determined at 412 nm. As a control drug, Tacrine (TAC) was used. The inhibition constants (Ki) were calculated by the Lineweaver–Burk plot [33]. All chemicals were purchased from Sigma-Aldrich Chemie GmbH (Hamburg, Germany).

### 2.4. CAs inhibition assay

Human CA isoforms (hCAI and hCAII) were purified by the Sepharose - 4B - L - tyrosine - sulfanilamide affinity segregation method as reported [34, 35]. Bradford technique was used to measure protein concentrations at 595 nm [36]. Inhibitory effects of the compounds were investigated by measuring the esterase activity according to Verpoorte et al. [37] as described in previous [38–40]. The hCA activity was determined by measuring the conversion of thep-nitrophenyl acetate substrate to *p* -nitro phenolate at 348 nm by the spectrophotometer (UV - VIS Spectrophotometer, UVmini-1240, Shimadzu Corporation, Kyoto, Japan) [41]. Acetazolamide (AZA) was used as a control drug. Lineweaver–Burk plot was used to calculate inhibition constants (Ki) of the compounds [33]. All chemicals were purchased from Sigma-Aldrich Chemie GmbH.

## 3. Results and discussion

### 3.1. Chemistry

This study reported the synthesis and bioactivities of the compounds having 1-(4-hydroxyphenyl)-2-((aryl)thio) ethanones chemical formula. 2-Bromo-4’-hydroxyacetophenone was attached to the heterocyclic rings via thioether functional group by conducting a single step reaction. The compounds 1, 2, 3, 5, and 6 were found as registered compounds at the Sci Finder database without any article and experimental data, while compound 4 [42] was reported as an intermediate. Therefore, the current study is the first study for the synthesis and bioactivities of 1, 2, 3, 5, and 6.

As a spectral evaluation, in ^1^H NMR, methylene protons were seen in the range of 5.07–4.49 ppm as expected. In some cases, the phenolic proton was not seen since it is an exchangeable proton. Signal of methyl substituent for the methyl-substituted compounds was seen in the range of 3.97–2.63 ppm. The carbonyl peak of the compounds in ^13^C NMR was seen in the range of 191.0–192.6 ppm. The carbon peak of the methylene bridge was seen at 49.1–41.1 ppm, while the carbon signal of methyl was in the range of 34.1–15.6 ppm. Further, calculated and measured m/z values of the compounds were also found compatible in HRMS analysis. In the IR spectra, C=O stretching band was recorded in the range of 1655–1672 cm^-1^.

### 3.2. Acetylcholinesterase inhibitory effects

In this study, the compounds 1–6 were screened against the AChE enzyme due to significant reports on AD of phenolic natural or synthetic compounds. IC50 and Ki values of the reference drug TAC were 40.76 nM (IC_50_) and 37.45 ±3.12 nM (Ki) towards AChE, as shown in Table. The compounds inhibited the AChE enzyme in nanomolar concentration in the range of Ki values of 22.13 ±1.96 - 62.11 ±6.00 nM and with IC_50_ values of 28.76–57.27 nM. The compounds 2 and 4 were found potent AChE inhibitors with the Ki values of 22.13 ±1.96 nM and 23.71 ±2.95 nM, respectively, while the compound 5 was the least inhibiting compound with the highest Ki value of 62.11 ±6.00 nM. On the other hand, the compound 1 was also considered as one of the potent inhibitors with the lowest IC_50_ value of 28.76 nM against AChE.

**Table:** Enzyme inhibitory results of the compounds 1–6.

Code	IC50 (nM)						Ki (nM)		
	hCA I	r^2^	hCA II	r^2^	AChE	r^2^	hCA I	hCA II	AChE
1	35.36	0.9743	29.36	0.9985	28.76	0.9781	14.60 ±2.06	8.76 ±0.84	26.53 ±5.43
2	32.84	0.9678	43.58	0.9771	32.23	0.9881	8.61 ±0.90	23.41 ±7.13	22.13 ±1.96
3	35.18	0.9975	39.83	0.9894	47.14	0.9900	17.76 ±2.08	31.64 ±3.29	37.79 ±11.41
4	48.13	0.9868	41.75	0.9955	46.20	0.9627	13.81 ±2.47	16.95 ±3.99	23.71 ±2.95
5	65.58	0.9928	33.97	0.9749	57.27	0.9863	42.59 ±7.59	14.32 ±5.10	62.11 ±6.00
6	62.54	0.9777	57.27	0.9775	47.47	0.9663	14.36 ±3.71	27.38 ±11.79	35.70 ±2.63
AZA*	23.90	0.9748	18.73	0.9890	-	-	21.74 ±5.48	18.27 ±3.56	-
TAC*	-	-	-	-	40.76	0.9877	-	-	37.45 ±3.12

*AZA: Acetazolamide; TAC: Tacrine; IC50: The half-maximal inhibitory concentration; Ki: Inhibition constant; hCA I, II: Human carbonic anhydrase I, II; AChE: Acetylcholinesterase; r^2^: The coefficient of determination; ±: Standard deviation; nM: Nanomolar.

The compounds 1 and 2 having a bicyclic ring showed considerable AChE inhibitory effects with low Ki values. When these two compounds compared with each other, it shows that oxygen atom was more favorable than the sulfur atom. Diazole (3) and tetrazole (6) derivatives were found more effective than triazole (5) derivative against the AChE enzyme. The compound 4 having 5-methyl-1,3,4-thiadiazol ring showed favorable enzyme inhibitory potency with Ki value of 23.71 ±2.95 nM in contrast to other five-membered compounds 3–6.

The compounds reported might be potential candidates for designing novel and more powerful AChE inhibitors for future studies. AD drugs such as donepezil, rivastigmine, galantamine have amine moiety in their chemical structure. So, the compounds having nitrogen atom/s may show favorable enzyme-ligand interactions [43]. Also, for the future concept, the most potent amine bearing phenolic compounds can be synthesized with Mannich reaction by the reaction of amine, formaldehyde, and phenolic compound under suitable reaction conditions as mono or bis Mannich bases against AChE based on our previous work regarding Mannich bases as promising AChE inhibitors [30].

### 3.3. Carbonic anhydrase inhibitory effects

Since the phenol group is an important zinc-binding group, the compounds 1–6 were evaluated towards CAs isoenzymes to show their CA inhibitory potency. The Table showed that the Ki values of reference drug AZA were 21.74 ±5.48 nM and 18.27 ±3.56 nM, whereas the IC_50_ values of AZA were 23.90 nM and 18.73 nM towards hCAI and II, respectively. Ki values of 1–6 were calculated as 8.61 ±0.90 – 42.59 ±7.59 nM (hCAI) and 8.76 ±0.84 – 31.64 ±3.29 nM (hCAII).

Among the compounds having the most common bicyclic rings, the compound 2 (Ki = 8.61 ±0.90 nM) was 2.5 fold more potent inhibitor against hCAI while the compound 1 (Ki = 8.76 ±0.84 nM) was 2.0 fold more potent against hCAII isoenzyme than reference AZA. On the other hand, among the compounds carrying five-membered rings 3–6, the compound 4 (Ki = 13.81 ±2.47 nM) for hCAI, and the compound 5 (Ki = 14.32±5.10 nM) for hCAII were potent CA inhibitors. When diazole (3), triazole (5) and tetrazole (6) derivatives were compared, the following results can be made. The tetrazole derivative 6 had good inhibitory potency against hCAI while triazole derivative 5 was effective inhibitor against hCAII isoenzyme. In series, benzothiazole and benzoxazole bearing compounds 1 and 2 were found the most potent CA inhibitor against the hCAs with the lowest Ki values. Also, it can be stated here that linking bicyclic ring with phenol function was found more rewarding modification than five-membered rings. Additional aromatic hydrophobic interactions with the active site residues of the enzyme may result in increasing enzyme inhibitory potency of these compounds. For future studies, these phenolic compounds can be used to synthesize novel CAIs by the reaction of phenol group with suitable reagents to obtain new sulfamate-based CAIs to see how this modification affects the CAs inhibition activity.

## 4. Conclusion

In this study, different heteroaryl mercapto compounds were combined with the phenolic group. The compounds 1-(4-hydroxyphenyl)-2-[(heteroaryl)thio]ethan-1-one 1–6 showed enzyme inhibitory potency at nanomolar concentrations. The compounds 2 and 4 were found potent AChE inhibitors with the Ki values of 22.13 ±1.96 nM and 23.71 ±2.95 nM, respectively, among others. On the other hand, based on the IC_50_ values, the compound 1 made attraction with the lowest IC_50_ value of 28.76 nM against AChE. The compounds 2 (Ki = 8.61 ±0.90 nM, on hCA I) and 1 (Ki = 8.76 ±0.84 nM, on hCA II) had also considerable CA inhibitory potency. The promising bioactivity results of these compounds can lead to the design of more potent enzyme inhibitors with additional molecular modifications for further studies.
